# Research on a Method of Locating Civil Aviation Radio Interference Sources Based on Time Difference of Arrival and Frequency Difference of Arrival for Four Unmanned Aerial Vehicles

**DOI:** 10.3390/s23187939

**Published:** 2023-09-16

**Authors:** Chao Zhou, Xingyu Zhu, Renhe Xiong, Kun Hu, Feng Ouyang, Chi Huang, Tao Huang

**Affiliations:** 1 Institute of Electronic and Electrical Engineering, Civil Aviation Flight University of China, Guanghan 618307, China; 15520664026@163.com; 2 College of Air Traffic Management, Civil Aviation Flight University of China, Guanghan 618307, China; zhcnzxy@gmail.com (X.Z.); renhexiong@gmail.com (R.X.); 17854335326@163.com (K.H.); 13778880943@163.com (C.H.); 3 CAAC Academy of Flight Technology and Safety, Civil Aviation Flight University of China, Guanghan 618307, China; oyf202313668304477@163.com

**Keywords:** four UAVs, civil aviation, radio interference source, time–frequency difference positioning

## Abstract

Monitoring and analyzing radio interference sources play a crucial role in ensuring the safe operation of civil aviation navigation, communication, airport management, and air traffic control. Traditional ground monitoring methods are slow and inadequate for tracking aerial and mobile interference sources effectively. Although flight methods such as helicopters and airships can effectively monitor aerial interference, the flight approval process is time-consuming and expensive. This paper investigates a novel approach to locating civil aviation radio interference sources using four unmanned aerial vehicles (UAVs) to address this issue. It establishes a model for aerial positioning of radio interference sources with the four UAVs and proposes a method for time synchronization and data communication among them. The paper conducts simulations of the four-UAV time–frequency difference positioning method, analyzing the geometric accuracy dilution with different deployment configurations of the UAVs, positioning biases, and root mean square errors (RMSEs) under varying interference source movement speeds. The simulation results provide crucial data to support subsequent experiments.

## 1. Introduction

In the realm of aviation, radio technology plays a critical role in communication, navigation, surveillance, meteorology, and various other aspects. However, the unauthorized establishment of “black radio”, “pseudo-base stations”, and similar devices has become a growing concern. These devices operate at frequencies perilously close to civil aviation radio frequencies, leading to escalating and severe interference with civil aviation communications. Such radio interference can significantly diminish air traffic control communications and crucial avionics equipment [1,2,3,4,5], posing a serious threat to aviation safety. Over the years, reported cases of radio interference at airports worldwide have shown a marked increase. For instance, according to the CAAC East China Regional Administration, there were 334 radio interference incidents in East China in 2015, and the number has remained consistently high, with over 300 incidents reported annually since then [6]. Additionally, the airspace near the airports of the Civil Aviation Flight University of China’s branches in Luoyang, Xinjin, and Suining has experienced multiple instances of radio interference, severely affecting normal flight training operations. In comparison to ground monitoring methods, air platform-based radio monitoring offers distinct advantages. Utilizing UAVs for monitoring circumvents airspace limitations and eliminates the complications and lengthy approval processes required for route clearance. Employing multiple UAVs provides several benefits, including heightened flexibility, increased positioning accuracy, extensive coverage, and rapid positioning. This approach proves particularly valuable in monitoring aerial and mobile interference sources, ultimately advancing the ranking of civil aviation radio interference sources.

In this paper, we present a novel approach for identifying the sources of civil aviation radio interference using a time–frequency difference positioning technique with four UAVs. By employing this method, we effectively mitigate the impact of multipath radio wave propagation caused by obstacles, while addressing the limitations of ground-based troubleshooting methods. The proposed method offers the capability not only to monitor aerial interference but also to effectively detect ground-to-air interference and ground interference. Furthermore, in comparison to existing unmanned aerial vehicle (UAV)-based methods for locating civil aviation interference sources, our approach demonstrates superior positioning accuracy, broader coverage area, faster positioning speed, and increased flexibility in tracking moving point targets.

## 2. Related Work

The concept of radio monitoring was initially introduced by Western countries, and subsequently, the Federal Aviation Administration (FAA) [7] implemented multiple fixed and relocatable stations to establish the Airport Radio Interference Monitoring System (AIMS) and Radio Interference Monitoring System (IMDS). The requirements for an interference source monitoring/direction finding system include a frequency coverage of 25–3000 MHz, a frequency scan rate of 1000 MHz/s, and a direction finding accuracy better than 2° [8]. In recent studies, various researchers proposed innovative techniques for different applications. Rakshit Ramesh et al. [9] proposed a new protocol and technique based on the time difference of arrival (TDOA) method for UAV positioning. Kilari et al. [10] introduced a linear programming initialization method to complement the TDOA algorithm. Mario Nicola et al. [2] presented a novel interference management concept capable of detecting intentional interference in navigation satellite system signals and determining its source. Sanat K Biswas et al. [11] explored the use of Kalman filters to efficiently geolocate and track dynamic and static RF interference sources based on real measurements from a geolocation system. Additionally, Adrien Perkins et al. [12] detailed the design, development, and flight testing of the JAGER visual navigation system. In China, radio monitoring networks are extensively employed to identify and exclude interference sources that may affect aviation, railroad, and telecommunication units. Notably, recent research by Hao Caiyong et al. [13] proposed a high-precision positioning method utilizing UAV assistance. Xu Bojian et al. [14] from Beijing Global Information Application Development Center performed an analysis based on radio frequency parameters and established a specimen database. Jin Ping et al. [15] from the School of Information Science and Engineering at Yanshan University introduced an improved MUSIC algorithm for localizing coherent interference cognitive users. Wang Guangyu [16] from the Technical Support Center of CAAC Northeast Regional Administration achieved precise positioning of radio interference sources. Additionally, Li Jinshan [17] from the School of Information Engineering and Automation at Kunming University of Science And Technology proposed an existing radio interference source positioning technique.

The positioning of radio wave sources plays a crucial role not only in military applications like electronic warfare but also in civilian domains such as navigation systems [18,19], internal security [20], and search and rescue missions [21,22]. The majority of methods analyzed in the literature apply to stationary interference sources. The potential of using widely available UAVs to enhance communication service quality and to extend coverage has been explored in the context of fifth-generation (5G) mobile networks and fixed interference source positioning systems, as presented in references [23,24,25], respectively. Among the more commonly used techniques for estimating the positions of mobile transmitters are TDOA and frequency difference of arrival (FDOA) measurements obtained from multiple sensors, as discussed in references [25,26]. In [27], target tracking techniques based on wireless sensor network (WSN) TDOA measurements are described. Sathyan et al. [28] also recommended the use of the extended Kalman filter (EKF). Similarly, Kim et al. [29] proposed a method involving correlated TDOA and Gaussian mixture. Kelner J M et al. [30] evaluated the effectiveness of signal Doppler frequency methods in locating mobile radiation sources using swarms of UAVs. The team led by Zhou Chao at the Civil Aviation Flight University of China (CAFUC) has undertaken extensive research in this field, constructing various UAV monitoring platforms dedicated to monitoring civil aviation radio interference sources [31,32,33,34,35,36,37,38,39].

The commonly employed techniques for localizing stationary radiation sources using UAV swarms primarily include the FDOA method, the positioning algorithm using a phase interferometer, the Dual Station Direction Finding (DF) cross-positioning algorithm, and the TDOA method for passive positioning techniques. Regarding the positioning of dynamic radiation sources by UAV swarms, the principal methods consist of the least squares method [40], spatial electromagnetic environment platform positioning [41], and radar positioning of dynamic radiation sources based on active positioning [42].

Although the time and angle positioning methods mentioned above can achieve a high level of positioning accuracy, they are constrained by the continuous operation of the target jammer and the complexity of the positioning algorithm. This paper introduces a fast and intuitive positioning method with a simple algorithm to determine the position of the interference source. In the designated measurement area, a matrix of several radio monitors is deployed, forming a radio monitoring network. This network continuously monitors the signal strength of the interference source within the area, measures the magnitude of its received power, and analyzes the received power magnitude data from the radio monitors. The proposed algorithm deduces the location of the interference source based on the data detected by the radio monitors, and its efficacy is validated through simulation.

## 3. Design of Four-UAV Time–Frequency Difference Positioning Method for Interference Sources

**Figure 1:**
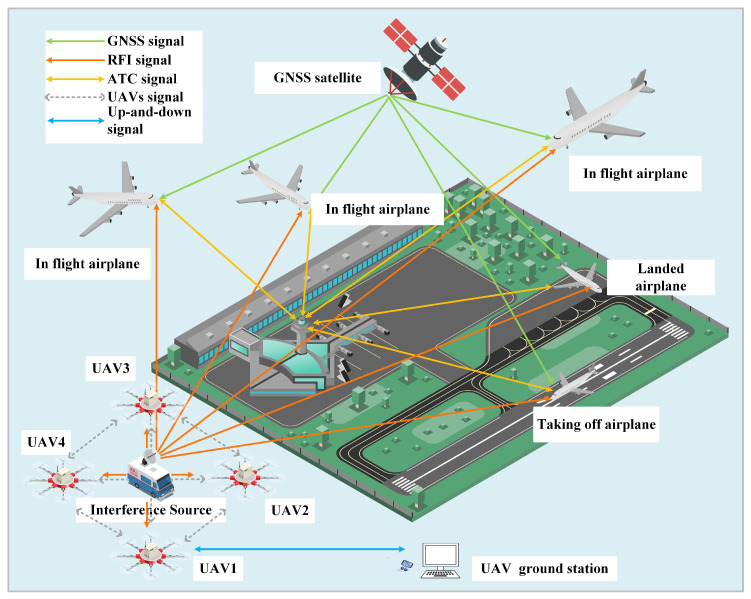
Four-UAV-based radio interference source positioning scenario for civil aviation.

The technique of cross-location between two UAVs primarily involves measuring the arrival angles between the interfering source and the monitoring station. The ray, originating from the monitoring station and passing through the interfering source, intersects with another ray to determine the position of the interference source. However, it should be noted that the cross-location method is only suitable for non-moving radio interference sources since the positioning accuracy is not sensitive to the positional errors of UAVs. In the case of a moving interference source, this method cannot provide precise positioning. Moreover, according to mathematical principles, the trajectory of a moving point with a constant difference in distances from two fixed points forms a hyperbola. To determine a point in three-dimensional space, at least three difference in distances and four monitoring stations are required. Therefore, TDOA positioning requires a minimum of four unmanned aerial vehicles (see Figure 1).

### 3.1. The Four-UAV Time–Frequency Difference Positioning Algorithm for Interference Sources

The four UAVs can be operated independently using their own paired remote controls or ground control computers. Once the UAVs have established a synchronized time reference, the signal-receiving equipment on each UAV monitoring platform measures the arrival time of the same interference source signal separately. Subsequently, the arrival time information, along with position, speed, and other relevant data from all UAVs, is collected and sent to the ground station through the downlink. The interference source’s position is then calculated using the TDOA–FDOA joint positioning method, and the location of the radio interference source is displayed on a map. The UAV ground station serves as the core component of the system, responsible for system control and data processing, enabling efficient cooperative control of the UAVs.

In the data processing of a multi-UAV cooperative monitoring network, it is essential to ensure that the measurement values provided by each UAV can be transformed into the same reference station coordinate system for analysis and expression. The geodetic coordinate system represents the position of the UAV in terms of longitude $L_{i}$, latitude $B_{i}$, and geodetic height $H_{i}$, i=1,2,…,P. In the Cartesian coordinate system, the position of the UAV is represented by variables $X_{i}$, $Y_{i}$, and $Z_{i}$. Given the geodetic coordinates of the UAV, the formula for calculating the Cartesian coordinates of UAV is as Equation (1):

(1) $$\left\{\begin{array}{c} x_{i}=\left(N+H_{i}\right)\cos B_{i}\cos L_{i}\\ y_{i}=\left(N+H_{i}\right)\cos B_{i}\sin L_{i}\\ z_{i}=\left[N\left(1-e_{1}^{2}\right)+H_{i}\right]\sin B_{i} \end{array}\right.$$

where $e_{1}$ represents the first eccentricity of the meridian ellipse and *N* denotes the curvature radius of the ellipsoidal surface along the prime vertical.

When the spatial Cartesian coordinate $U_{i}\left(x_{i},y_{i},z_{i}\right)$ of a UAV is known, the formula for calculating the geodetic coordinates of UAV is as Equation (2).

(2) $$\left\{\begin{array}{c} L_{i}=\arctan\frac{y_{i}}{x_{i}}\\ B_{i}=\arctan\frac{\left(N+H_{i}\right)z_{i}}{\left(N+H_{i}-e_{1}^{2}N^{2}\right)\sqrt{x_{i}^{2}+y_{i}^{2}}}\\ H_{i}=\frac{\sqrt{x_{i}^{2}+y_{i}^{2}}}{\cos B_{i}}-N \end{array}\right.$$

Let $R={\left[x,y,z\right]}^{T}$ represent the position of the moving civil aviation radio interference source and $\dot{R}={\left[\dot{x},\dot{y},\dot{z}\right]}^{T}$ denote its moving speed. There are a total of four UAVs with their flight positions described in spatial Cartesian coordinates as $U_{i}=\left[x_{i},y_{i},z_{i}\right]$ and their flight speeds as ${\dot{U}}_{i}=\left[{\dot{x}}_{i},{\dot{y}}_{i},{\dot{z}}_{i}\right]$, i=1,2,…,4. Considering UAV $U_{1}$ as the master station, the distance difference, known as the range difference of arrival (RDOA) between the remaining UAVs and the master station concerning the mobile interference source can be expressed using Equation (3) as follows:

(3) $$r_{i,1}=d_{i}-d_{1}+n_{i,1}$$

where $d_{i}={∥R-U_{i}∥}_{2}=\sqrt{{\left(x-x_{i}\right)}^{2}+{\left(y-y_{i}\right)}^{2}+{\left(z-z_{i}\right)}^{2}}$ represents the actual distance between the *i*-th UAV and the mobile interference source and $n_{i,1}$ denotes the measurement error of the *i*-th time.

Expand Equation (3) as follows:

(4) $$2{\left(U_{i}-U_{1}\right)}^{T}R+2r_{i,1}d_{1}=\left(U_{i}^{T}U_{i}-U_{1}^{T}U_{1}-r_{i,1}^{2}\right)+2d_{i}n_{i,1}$$

The time derivative of Equation (4) allows us to utilize the relevant information of FDOA:

(5) $$\begin{array}{cc} \; & 2{\left({\dot{U}}_{i}-{\dot{U}}_{1}\right)}^{T}R+2{\left(U_{i}-U_{1}\right)}^{T}\dot{R}+2r_{i,1}{\dot{d}}_{1}+2{\dot{r}}_{i,1}d_{1}\\ \; & =2\left({\dot{U}}_{i}^{T}U_{i}-{\dot{U}}_{1}^{T}U_{1}-r_{i,1}{\dot{r}}_{i,1}\right)+2d_{i}{\dot{n}}_{i,1}+2{\dot{d}}_{i}n_{i,1} \end{array}$$

The time derivative of the actual distance $d_{i}={∥R-U_{i}∥}_{2}$ between the *i*-th UAV and the mobile interference source is represented as Equation (6):

(6) $${\dot{d}}_{i}=\frac{\left({\dot{U}}_{i}-{\dot{U}}_{1}\right)\left(U_{i}-U_{1}\right)}{d_{i}}$$

Based on the monitoring data, the TDOA information of the three UAVs is represented by $T_{d}=\left[t_{2,1},t_{3,1},t_{4,1}\right]$, the FDOA information is denoted by $F_{d}=\left[f_{2,1},f_{3,1},f_{4,1}\right]$, their RDOA is indicated as $r=\left[r_{2,1},r_{3,1},r_{4,1}\right]$ and the rate of change in RDOA is expressed as $\dot{r}=\left[{\dot{r}}_{2,1},{\dot{r}}_{3,1},{\dot{r}}_{4,1}\right]$. The relationship between these parameters can be expressed as Equation (7):

(7) $$\left\{\begin{array}{c} r=c\times T_{d}=d+n\\ \dot{r}=c\times \frac{F_{d}}{f_{0}}=\dot{d}+\dot{n} \end{array}\right.$$

where $f_{0}$ represents the carrier frequency; *c* denotes the transmission speed of the radio interference source frequency; and $n=\left[n_{2,1},n_{3,1},n_{4,1}\right]$ and $\dot{n}=\left[{\dot{n}}_{2,1},{\dot{n}}_{3,1},{\dot{n}}_{4,1}\right]$ refer to the time noise vector and the frequency noise vector, respectively. In this algorithm, the noises are temporarily assumed to be zero-mean Gaussian white noise.

In the first step, we estimate by combining Equations (4) and (5), resulting in the following:

(8) $$Q_{1}\theta_{1}-h_{1}=\varepsilon_{1}$$

where $\theta_{1}={\left[R^{T},R_{1},{\dot{R}}^{T},{\dot{R}}_{1}\right]}_{8\times 1}^{T}$, $B=2\times \mathrm{diag}\left(\left[d_{2},d_{3},d_{4}\right]\right)$, $\dot{B}=2\times \mathrm{diag}\left(\left[{\dot{d}}_{2},{\dot{d}}_{3},{\dot{d}}_{4}\right]\right)$, $h_{1}={\left[\begin{array}{c} U_{2}^{T}U_{2}-U_{1}^{T}U_{1}-r_{2,1}^{2}\\ \cdots \\ U_{4}^{T}U_{4}-U_{1}^{T}U_{1}-r_{4,1}^{2}\\ 2\left({\dot{U}}_{2}^{T}U_{2}-{\dot{U}}_{1}^{T}U_{1}-{\dot{r}}_{2,1}r_{2,1}\right)\\ \cdots \\ 2\left({\dot{U}}_{4}^{T}U_{4}-{\dot{U}}_{1}^{T}U_{1}-{\dot{r}}_{M,1}r_{M,1}\right) \end{array}\right]}_{6\times 1}$, $Q_{1}={\left[\begin{array}{cccc} {\left(U_{2}-U_{1}\right)}^{T} & r_{2,1} & 0_{1\times 3} & 0\\ \ldots & \cdots & \cdots & \cdots \\ {\left(U_{4}-U_{1}\right)}^{T} & r_{4,1} & 0_{1\times 3} & 0\\ {\left({\dot{U}}_{2}-{\dot{U}}_{1}\right)}^{T} & {\dot{r}}_{2,1} & {\left(U_{2}-U_{1}\right)}^{T} & r_{2,1}\\ \cdots & \cdots & \cdots & \cdots \\ {\left({\dot{U}}_{4}-{\dot{U}}_{1}\right)}^{T} & {\dot{r}}_{4,1} & {\left(U_{4}-U_{1}\right)}^{T} & r_{4,1} \end{array}\right]}_{6\times 8}$, $\varepsilon_{1}=B_{1}\Delta \eta =\left[\begin{array}{cc} B & 0_{3\times 3}\\ \dot{B} & B \end{array}\right]\left[\begin{array}{c} n\\ \dot{n} \end{array}\right]$.

Based on the preceding matrix, Equation (8) is transformed to the following:

(9) $$\theta_{1}={\left(Q_{1}^{T}T_{1}Q_{1}\right)}^{-1}Q_{1}^{T}T_{1}h_{1}$$

The obtained Equation (9) represents the weighted least squares estimate of the first step, where $T_{1}={\left(B_{1}Z^{-1}B_{1}^{T}\right)}^{-1}$, *Z* denote the covariance matrix of the measurement noise Δη.

(10) $$\mathrm{cov}\left(\theta_{1}\right)={\left(Q_{1}^{T}T_{1}Q_{1}\right)}^{-1}$$

In the second-step estimate, the time–frequency difference joint positioning algorithm has two constraints on distance:

(11) $${\left(R-U_{1}\right)}^{T}\left(R-U_{1}\right)=d_{1}^{2}$$

(12) $${\left(\dot{R}-{\dot{U}}_{1}\right)}^{T}\left(R-U_{1}\right)={\dot{d}}_{1}d_{1}$$

Based on Equations (11) and (12), we can establish the following constraint model:

(13) $$Q_{2}\theta_{2}-h_{2}=\varepsilon_{2}$$

Let $\theta_{1,R}={\left[\theta_{1}\left(1\right),\theta_{1}\left(2\right),\theta_{1}\left(3\right)\right]}^{T}$, $\theta_{1,\dot{R}}={\left[\theta_{1}\left(5\right),\theta_{1}\left(6\right),\theta_{1}\left(7\right)\right]}^{T}$, $\theta_{2}=\left[\begin{array}{c} \left(R-U_{1}\right)⊙\left(R-U_{1}\right)\\ \left(\dot{R}-U_{1}\right)⊙\left(R-U_{1}\right) \end{array}\right]Q_{2}=\left[\begin{array}{cc} I_{3\times 3} & 0_{3\times 3}\\ 1_{1\times 3} & 0_{1\times 3}\\ 0_{3\times 3} & I_{3\times 3}\\ 0_{1\times 3} & 1_{1\times 3} \end{array}\right]$, $h_{2}=\left[\begin{array}{c} \left(\theta_{1,R}-U_{1}\right)⊙\left(\theta_{1,R}-U_{1}\right)\\ \theta_{1}{\left(4\right)}^{2}\\ \left(\theta_{1,\dot{R}}-U_{1}\right)⊙\left(\theta_{1,R}-U_{1}\right)\\ \theta_{1}\left(8\right)\theta_{1}\left(4\right) \end{array}\right]$, where $I_{3\times 3}$ is a 3 × 3 identity matrix, $0_{3\times 3}$ is a 3 × 3 zero matrix, $1_{1\times 3}$ is a 1 × 3 matrix with all ones, $0_{1\times 3}$ is a 1 × 3 zero matrix, and ⊙ represents the product of vectors.

Based on the preceding matrix, Equation (13) is transformed to the following:

(14) $$\theta_{2}={\left(Q_{2}^{T}T_{2}Q_{2}\right)}^{-1}Q_{2}^{T}T_{2}h_{2}$$

The obtained Equation (14) represents the weighted least squares estimation of the second step, where $T_{2}={\left(B_{2}\mathrm{cov}\left(\theta_{1}\right)B_{2}^{T}\right)}^{-1}$, $B_{2}=\left[\begin{array}{cccc} 2\mathrm{diag}\left(R-U_{1}\right) & 0 & 0_{3\times 3} & 0\\ 0_{1\times 3} & 2d_{1} & 0_{1\times 3} & 0\\ \mathrm{diag}\left(\dot{R}-{\dot{U}}_{1}\right) & 0 & \mathrm{diag}\left(R-U_{1}\right) & 0_{3\times 1}\\ 0_{1\times 3} & d_{1} & 0_{1\times 3} & {\dot{d}}_{1} \end{array}\right]$.

Estimate $\theta_{2}$ and utilize Equations (15) and (16) to calculate the position information and velocity information of the mobile interference source:

(15) $$R=V{\left[\sqrt{\theta_{2}\left(1\right)},\sqrt{\theta_{2}\left(2\right)},\sqrt{\theta_{2}\left(3\right)}\right]}^{T}+U_{1}$$

(16) $$\dot{R}=V{\left[\frac{\theta_{2}\left(4\right)}{\sqrt{\theta_{2}\left(1\right)}},\frac{\theta_{2}\left(5\right)}{\sqrt{\theta_{2}\left(2\right)}},\frac{\theta_{2}\left(6\right)}{\sqrt{\theta_{2}\left(3\right)}}\right]}^{T}+{\dot{U}}_{1}$$

where $V=\mathrm{diag}\left(\mathrm{sgn}\left(\theta_{1,R}-U_{1}\right)\right)$, where sgn is the positive or negative sign.

The first-step estimation and the second-step estimation mentioned above are repeated in a cycle until the difference between the two estimation results is smaller than the predefined threshold or the number of cycles reaches the set limit. At that point, the cycle is terminated, and the final result at the end of the cycle represents the most accurate estimate obtained using the algorithm.

Accordingly, the overall flow of the four-UAV time–frequency difference positioning interference source algorithm can be obtained: (See Algorithm 1).
**Algorithm 1:** The overall flow of the four-UAV time–frequency difference positioning interference sourceStep 1: Input the UAV position and velocity information, followed by a conversion of the geodetic coordinates of the UAV into spatial Cartesian coordinates using a coordinate system transformation model specifically tailored for the algorithm;Step 2: Construct a joint four-UAV TDOA–FDOA positioning model ($Q_{1}$ and $h_{1}$), and let $T_{1}$ be the identity matrix;Step 3: Calculate the weighted least squares estimate for the first step based on the localization model: $\theta_{1}={\left(Q_{1}^{T}T_{1}Q_{1}\right)}^{-1}Q_{1}^{T}T_{1}h_{1}$;Step 4: Calculate the coefficient matrix $B_{1}$ from $\theta_{1}$, and reconstruct the weight matrix: $T_{1}={\left(B_{1}Z^{-1}B_{1}^{T}\right)}^{-1}$;Step 5: Repeat steps 3 and 4 until the absolute difference between the two results is smaller than the predefined threshold or the set number of cycles is reached. At this point, terminate the cycle, and obtain $\mathrm{cov}\left(\theta_{1}\right)={\left(Q_{1}^{T}T_{1}Q_{1}\right)}^{-1}$;Step 6: The constraint models for the joint four-UAV TDOA–FDOA positioning are constructed based on $\theta_{1}$ ($Q_{2}$ and $h_{2}$);Step 7: Construct the coefficient matrix $B_{2}$ of the constraint model weight matrix, and calculate the weight matrix: $T_{2}={\left(B_{2}\mathrm{cov}\left(\theta_{1}\right)B_{2}^{T}\right)}^{-1}$;Step 8: Calculate the weighted least squares estimate for the second step based on the constrained model: $\theta_{2}={\left(Q_{2}^{T}T_{2}Q_{2}\right)}^{-1}Q_{2}^{T}T_{2}h_{2}$;Step 9: Calculate *R* and $\dot{R}$ for mobile radio interference sources based on $\theta_{2}$;Step 10: Repeat steps 6 to 8 until the absolute difference between the two results is smaller than the predefined threshold or the set number of cycles is reached. At this point, terminate the cycle and obtain the final calculated interference sources *R* and $\dot{R}$;Step 11: Transform the spatial Cartesian coordinates of the radio interference source into geodetic coordinates using the coordinate system conversion model and then output them.

### 3.2. Design of Four-UAV Time Synchronization

**Figure 2:**
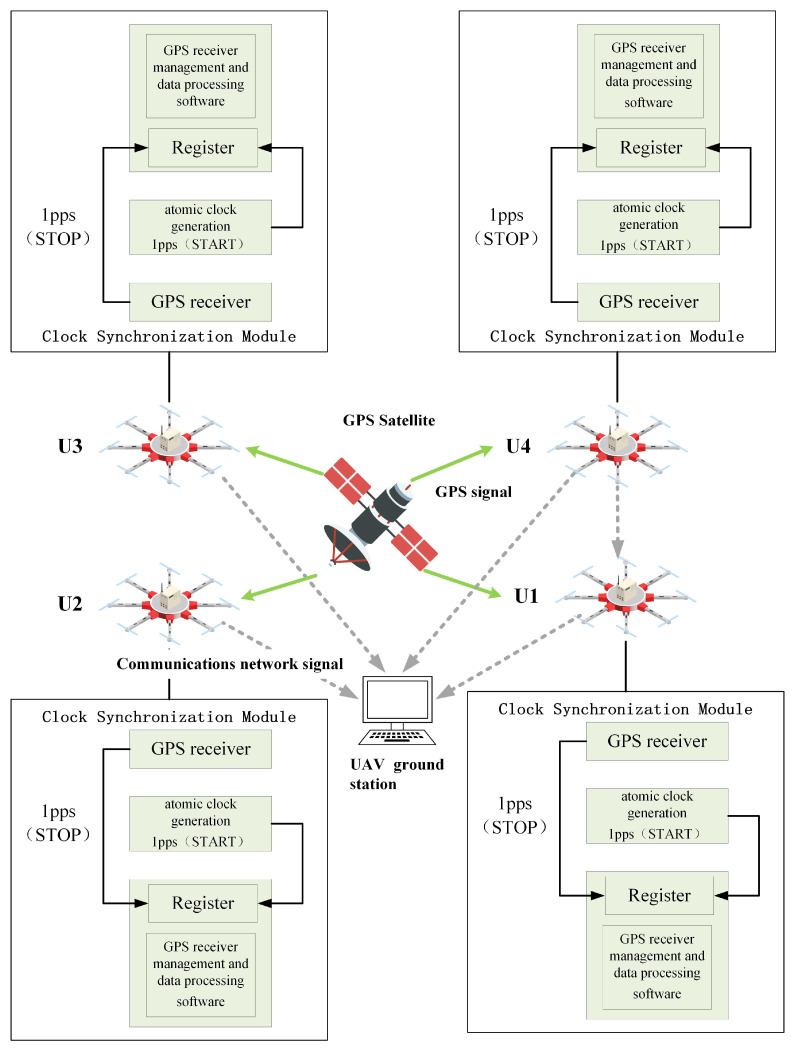
Schematic diagram of the four UAVs’ time synchronization settings.

Due to the different clock behaviors on each UAV, ensuring meaningful time measurements necessitates adopting one UAV’s time as the standard and synchronizing the time of the other three UAVs with it. In this paper, the utilized clock synchronization method involves transmitting the time difference between all UAVs and the GPS time to the ground station. Following processing using the time–frequency synchronization algorithm, the time difference information is inputted into the time–frequency difference positioning algorithm. Figure 2 depicts the schematic diagram of the four UAVs’ time synchronization settings.

Let the clock time of UAV $U_{1}$ be $t_{U_{1}}$, the clock time of UAV $U_{2}$ be $t_{U_{2}}$, the clock time of UAV $U_{3}$ be $t_{U_{3}}$ and the clock time of UAV $U_{4}$ be $t_{U_{4}}$, while the GPS time is denoted as $t_{GPS}$. The measurement method for the clock difference of the four UAVs is as follows: Under the same co-viewing schedule constraint, the GPS receivers of the four UAVs simultaneously receive the same GPS satellite signal. The output of the GPS receivers of all four UAVs produces a GPS time second pulse, which is then transmitted to the built-in counter of the GPS receivers, resulting in the GPS on-star time. By subtracting the received GPS on-star time from the local atomic clock seconds signal generated by the clock synchronization module, along with the time delays of the UAV monitoring platform equipment and the GPS signal reaching the UAV monitoring platform, we obtain the difference between the clock time of each UAV and the GPS on-star time. This process is executed by the GPS receiver management and data processing software within the clock synchronization module. The resulting difference data are then transmitted from the UAVs to the ground for further processing. Consequently, subtracting the air–ground signal transmission delay from this difference yields the clock difference between the ground clock and the GPS clock.

(17) $$\Delta t_{iGPS}=t_{i}-t_{GPS}-t_{Ri}-\tau_{i}-\kappa_{i}\;\left(i=U_{1},U_{2},U_{3},U_{4}\right)$$

where $t_{i}$ represents the local time generated by the clock synchronization module of each UAV platform when the GPS signal is received by the UAV; $t_{GPS}$ is the on-star time of the GPS satellite transmit signal; $t_{Ri}$ denotes the equipment time delay of the corresponding UAV platform; $\tau_{i}$ is the transmission time delay of the GPS satellite signal reaching the UAV; and $\kappa_{i}$ is the ground-to-air signal transmission time delay and is given by the following:

(18) $$\tau_{i}=\frac{\sqrt{{\left(x-x_{i}\right)}^{2}+{\left(y-y_{i}\right)}^{2}+{\left(z-z_{i}\right)}^{2}}}{c}\;\left(i=U_{1},U_{2},U_{3},U_{4}\right)$$

where (x,y,z) represents the Cartesian coordinates of the GPS satellite and $\left(x_{i},y_{i},z_{i}\right)$ denotes the Cartesian coordinates of each UAV. By utilizing Equations (17) and (18), it is possible to calculate the difference between the local clock of the four UAVs and the GPS clock at the ground station, resulting in the values of $\Delta t_{U_{1},GPS}$, $\Delta t_{U_{2},GPS}$, $\Delta t_{U_{3},GPS}$ and $\Delta t_{U_{4},GPS}$. By subtracting the values of $\Delta t_{U_{1},GPS}$ from $U_{2},U_{3},U_{4}$ and $U_{1}$, we obtain the differences $\Delta t_{U_{1},U_{2}}$, $\Delta t_{U_{1},U_{3}}$, and $\Delta t_{U_{1},U_{4}}$ between the clocks of the three UAVs $U_{2},U_{3},$ and $U_{4}$ and that of UAV $U_{1}$.

(19) $$\begin{array}{cc} \Delta t_{U_{1},U_{2}} & =t_{U_{1}}-t_{U_{2}}\\ \; & =\left(\Delta t_{U_{1},GPS}+\Delta t_{GPS}+\tau_{U_{1}}+t_{RU_{1}}+\kappa_{U_{1}}\right)-\left(\Delta t_{U_{2},GPS}+\Delta t_{GPS}+\tau_{U_{2}}+t_{RU_{2}}+\kappa_{U_{2}}\right)\\ \; & =\left(\Delta t_{U_{1},GPS}-\Delta t_{U_{2},GPS}\right)+\left(\tau_{U_{1}}-\tau_{U_{2}}\right)+\left(t_{RU_{1}}-t_{RU_{2}}\right)+\left(\kappa_{U_{1}}-\kappa_{U_{2}}\right) \end{array}$$

(20) $$\begin{array}{cc} \Delta t_{U_{1},U_{3}} & =t_{U_{1}}-t_{U_{3}}\\ \; & =\left(\Delta t_{U_{1},GPS}+\Delta t_{GPS}+\tau_{U_{1}}+t_{RU_{1}}+\kappa_{U_{1}}\right)-\left(\Delta t_{U_{3},GPS}+\Delta t_{GPS}+\tau_{U_{3}}+t_{RU_{3}}+\kappa_{U_{3}}\right)\\ \; & =\left(\Delta t_{U_{1},GPS}-\Delta t_{U_{3},GPS}\right)+\left(\tau_{U_{1}}-\tau_{U_{3}}\right)+\left(t_{RU_{1}}-t_{RU_{3}}\right)+\left(\kappa_{U_{1}}-\kappa_{U_{3}}\right) \end{array}$$

(21) $$\begin{array}{cc} \Delta t_{U_{1},U_{4}} & =t_{U_{1}}-t_{U_{4}}\\ \; & =\left(\Delta t_{U_{1},GPS}+\Delta t_{GPS}+\tau_{U_{1}}+t_{RU_{1}}+\kappa_{U_{1}}\right)-\left(\Delta t_{U_{4},GPS}+\Delta t_{GPS}+\tau_{U_{4}}+t_{RU_{4}}+\kappa_{U_{4}}\right)\\ \; & =\left(\Delta t_{U_{1},GPS}-\Delta t_{U_{4},GPS}\right)+\left(\tau_{U_{1}}-\tau_{U_{4}}\right)+\left(t_{RU_{1}}-t_{RU_{4}}\right)+\left(\kappa_{U_{1}}-\kappa_{U_{4}}\right) \end{array}$$

In this way, the three UAVs $U_{2},U_{3},$ and $U_{4}$ can be synchronized on time based on the reference of the $U_{1}$ UAV’s clock.

### 3.3. Design of Four UAVs’ Data Communication

The communication system for monitoring civil aviation radio interference sources using four UAVs can be divided into three main parts: inter-aircraft link communication, UAV platform to ground station link communication (downlink), and ground station to UAV platform link communication (uplink). The inter-aircraft link is established based on a UAV self-assembling network architecture. All four UAVs are equipped with self-assembling network communication radios, enabling interaction and information exchange among them. Through the inter-aircraft link, each UAV shares its position, speed, and other relevant information with the other UAVs. For the downlink communication, data transmission radios are utilized. Each UAV is equipped with an antenna and a hardware front-end for the Software Defined Radio (SDR) platform, known as the Universal Software Radio Peripheral N321 (USRP N321). This setup enables the UAVs to receive signals from radio interference sources. The received signals are then processed and transmitted to the ground station via data radios. In addition, the four UAVs transmit their collected information to the ground station using data radios. The uplink communication relies on wireless self-assembling radios. Each of the four UAVs is equipped with a self-assembling radio, similar to the inter-aircraft communication equipment. These radios receive control commands from the ground self-assembling transmitter radio, which is connected to a laptop computer functioning as the ground station. The laptop runs the necessary software for UAV flight, facilitating communication between UAVs, between UAVs and ground stations, and between ground stations and UAVs.

**Figure 3:**
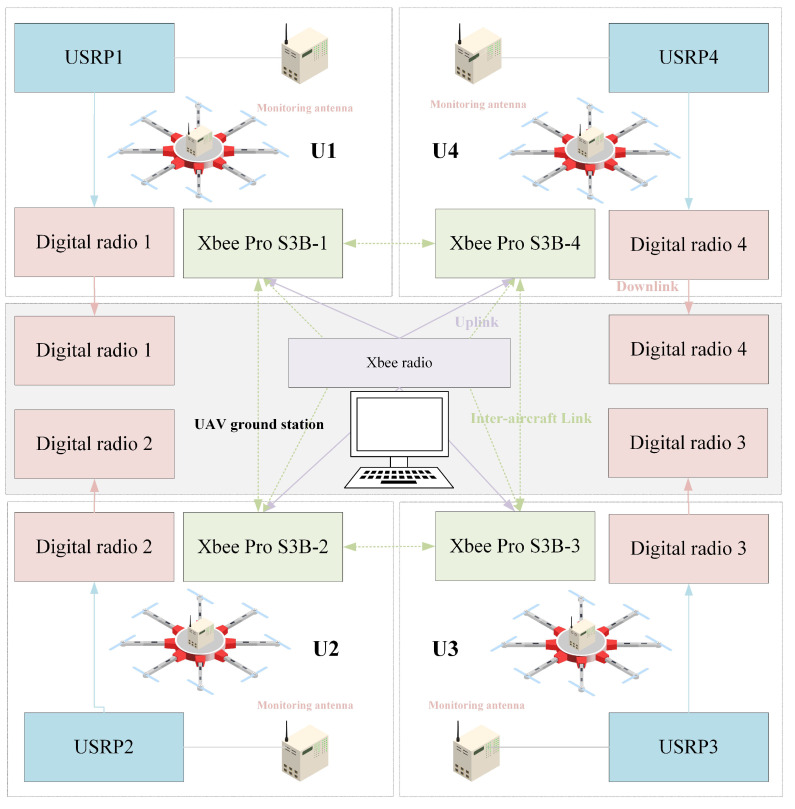
Schematic diagram of four UAVs’ data communication design.

Figure 3 depicts the schematic design of the four UAVs’ data communication. For the downlink design, each UAV is equipped with a digital transmission radio responsible for the real-time transmission of both the UAV’s flight information and the USRP-converted digital signal information to the digital reception radio. This arrangement enables the ground station to display the radio spectrum monitored by the UAV platform and facilitates the monitoring of the UAV’s flight status.

The flight control system of UAV is based on the open-source hardware and software Pixhawk 2.4.8, utilizing the Mavlink communication protocol. The design of the inter-aircraft link and downlink is as follows: For the uplink, the on-board self-assembling device is the Xbee Pro S3B radio, which operates using the DIGI mesh protocol and is configured as a routing model. This setup allows it to receive control commands from the ground station and facilitates data interaction among the four UAVs. As for the ground device, the Xbee radio is chosen and configured as a coordinator. The Mavlink protocol is nested within the outer mesh protocol to enable the ground station to send flight control commands to the UAVs.

To ensure a smooth communication link, it is essential to appropriately reduce the communication load. As a result, the uplink currently transmits only UAV control command packets and UAV-desired position packets. The inter-aircraft link, on the other hand, solely requires UAV flight position data packets. Lastly, the downlink necessitates only the transmission of UAV flight position packages, UAV flight attitude packages, UAV flight status packages, and radio spectrum packages.

## 4. Simulation of Four-UAV Time–Frequency Difference Positioning Model for Interference Sources

We have set up simulated interference sources within the university premises, with a frequency set around 442 MHz. The joint positioning method for civil aviation radio interference sources based on four UAVs was implemented using the MATLAB program. The simulation involved analyzing the UAV deployment configurations and different moving speeds of radio interference sources independently.

### 4.1. Positioning Performance of UAVs with Different Deployment Configurations

According to the star, flat rhombus, inverted triangle, and parallelogram deployment patterns, four UAVs were set up with their respective spatial Cartesian coordinate systems. A GDOP positioning accuracy analysis was conducted for each deployment pattern, and the specified UAV coordinates can be found in Table 1.

**Figure 4:**
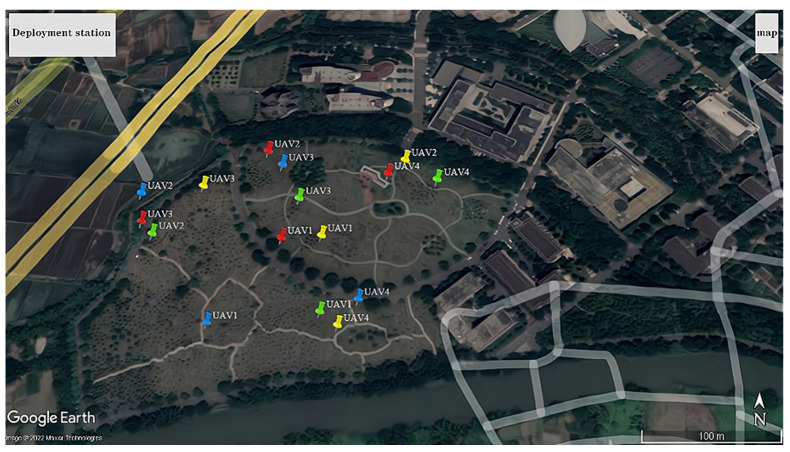
Diagram of UAV deployment configurations.

The yellow markers represent the star deployment configuration, the red markers represent the flat rhombus deployment configuration, the green markers represent the inverted triangle deployment configuration, and the blue markers represent the parallelogram deployment configuration, as shown in Figure 4.

The measurement time error is set to 10 ns, and the UAV station error is set to 5 m. The interference source is considered a fixed source, and the observation ranges are denoted as x=−200km∼200km, y=−200km∼200km, with the target height as z=10km. In the spatial Cartesian coordinate system, interference source positions are randomly generated. Each interference source location is traversed, and the GDOP is calculated for UAV positioning.

**Figure 5:**
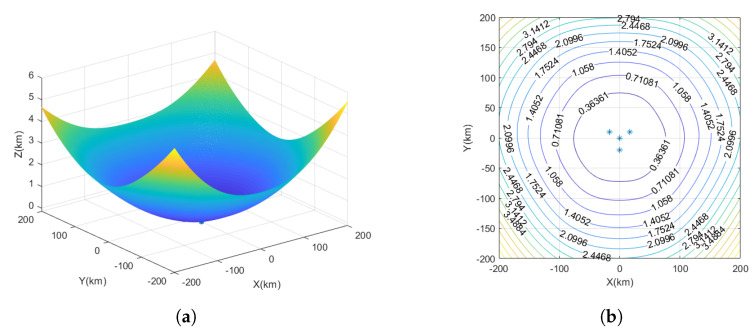
The GDOP of UAV star deployment configuration. (**a**) Three-dimensional plot of positioning error for star deployment configuration. (**b**) The GDOP of star deployment configuration.

Based on Figure 5a, the positioning errors of the star deployment configuration in the vertical dimension can be observed. From Figure 5b, it can be seen that when using star deployment configuration, the time–frequency difference positioning method has a balanced performance in three-dimensional space, with UAV1 as the center, and the farther the interference source is from UAV1, the larger the positioning error is. The position of the interference source is about 75 km from the position of UAV1, and the positioning error is about 360 m; the position of the interference source is about 100 km from the position of UAV1, and the positioning error is about 700 m; and the position of the interference source is about 120 km from the position of UAV1, and the positioning error is more than 1 km.

**Figure 6:**
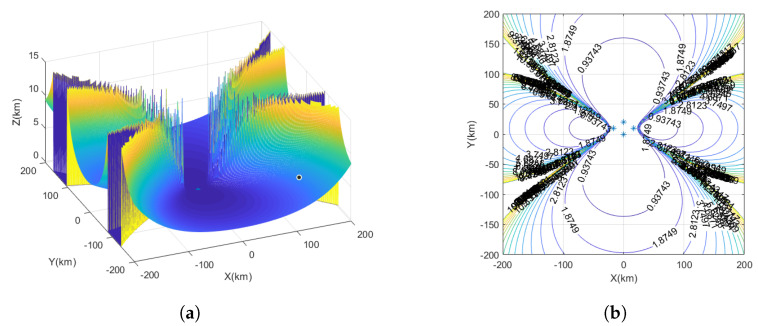
The GDOP of UAV flat rhombus deployment configuration. (**a**) Three-dimensional plot of positioning error for flat rhombus deployment configuration. (**b**) The GDOP of flat rhombus deployment configuration.

Based on Figure 6a, the positioning errors of the flat rhombus deployment configuration in the vertical dimension can be observed. Figure 6b illustrates the performance of the time–frequency difference positioning method in three-dimensional space using a flat rhombus deployment configuration with UAV1 as the center. The results show a relatively balanced performance, but there is a notable monitoring blind area. As the interference source moves farther away from UAV1, the range of this blind area increases. Moreover, the positioning performance of the interference source in the vertical direction of UAV1 is better than that in the horizontal direction. At approximately 40 km from the horizontal direction of UAV1, the positioning error of the interference source location is approximately 930 m. At the same positioning error, the distance from the vertical direction of UAV1 is approximately 140–160 km.

**Figure 7:**
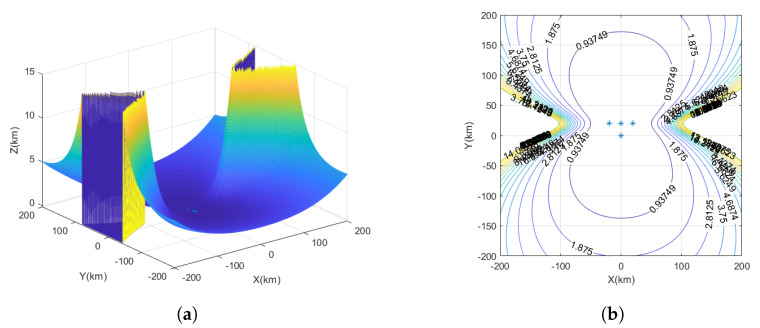
The GDOP of UAV inverted triangle deployment configuration. (**a**) Three-dimensional plot of positioning error for inverted triangle deployment configuration. (**b**) The GDOP of inverted triangle deployment configuration.

Based on Figure 7a, the positioning errors of the inverted triangle deployment configuration in the vertical dimension can be observed. Figure 7b presents the performance analysis of the time–frequency difference positioning method in three-dimensional space, employing an inverted triangle deployment configuration with UAV1 positioned at the center. The results demonstrate a relatively balanced performance; however, there is a significant monitoring blind area when the interference source is located 100 km away from the horizontal direction of UAV1. Furthermore, as the interference source moves farther away from UAV1, the range of this monitoring blind area expands. Moreover, the positioning performance of the interference source in the vertical direction of UAV1 surpasses that in the horizontal direction. At a distance of approximately 50 km from the horizontal direction of UAV1, the positioning error for the interference source location is approximately 930 m. At the same positioning error, the distance from the vertical direction of UAV1 is approximately 140–170 km.

**Figure 8:**
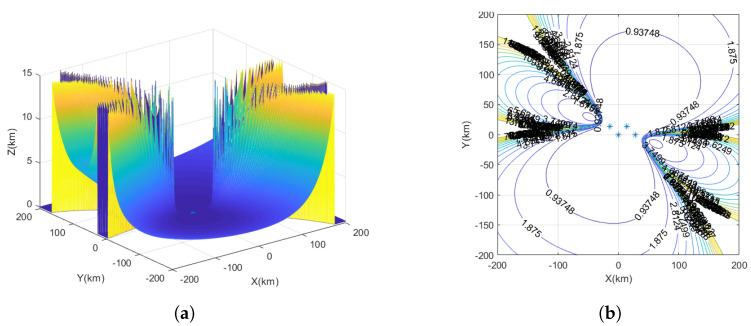
The GDOP of UAV parallelogram deployment configuration. (**a**) Three-dimensional plot of positioning error for parallelogram deployment configuration. (**b**) The GDOP of parallelogram deployment configuration.

Based on Figure 8a, the positioning errors of the parallelogram deployment configuration in the vertical dimension can be observed. Figure 8b illustrates the performance of the time–frequency difference positioning method in three-dimensional space using a parallelogram deployment configuration with UAV1 as the center. The results indicate a relatively balanced performance; however, a monitoring blind area is present. Notably, as the interference source moves farther away from UAV1, the range of this monitoring blind area expands. Moreover, the positioning performance of the interference source in the vertical direction of UAV1 outperforms that in the horizontal direction. The positioning error of the interference source location is approximately 930 m at distances ranging from about 40 km to 55 km from the horizontal direction of UAV1. For the same positioning error, the distance from the vertical direction of UAV1 is approximately 150 km to 160 km.

Through the GDOP analysis of the four UAV deployment configurations, it is evident that the star-based deployment configuration exhibits a balanced positioning performance with smaller errors compared to other deployment configurations. When employing the UAV platform for monitoring, the star-based formation flight yields comprehensive positioning coverage and high positioning accuracy.

### 4.2. Interference Source Positioning Performance at Different Moving Speeds

Assuming fixed initial coordinates and speeds for the four UAVs, Table 2 presents their respective coordinates and speed information. The initial position of interference source is R(280,320,270), and its speed is set at ${\dot{R}}_{1}\left(0,0,0\right)$, ${\dot{R}}_{1}\left(20,0,0\right)$, ${\dot{R}}_{1}\left(40,0,0\right)$, and ${\dot{R}}_{1}\left(80,0,0\right)$. To analyze the positioning performance, we utilize the time–frequency difference positioning algorithm through 5000 Monte Carlo simulations. The TDOA measurement value is subject to noise variance levels $10\log\left(c^{2}\sigma_{d}^{2}\right)$ ranging from −20 to 20, while the FDOA measurement value experiences noise variance levels set at 0.1 times that of TDOA. We evaluate the positioning performance using both bias and RMSE.

**Figure 9:**
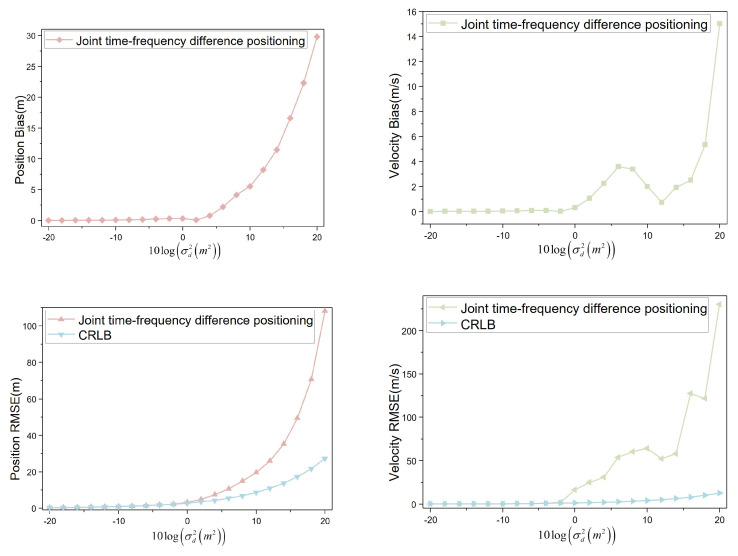
Positioning bias and RMSE (${\dot{R}}_{1}\left(0,0,0\right)$).

When the moving speed of the interference source is set to ${\dot{R}}_{1}\left(0,0,0\right)$, indicating the interference source is stationary, the four UAVs conduct aerial monitoring at the speeds specified in Table 2. The obtained simulation data were visualized to analyze the positioning bias and RMSE variation trends, as shown in Figure 9.

The speed of the interference source is ${\dot{R}}_{1}\left(0,0,0\right)$). The position bias remains below 1 m when the noise variance level is not greater than 4. For noise variance levels ranging from −20 to 4, the position bias shows minimal changes. However, the position bias is greater than 1 m when the noise variance level is greater than 4. The position bias changes more when the noise variance level is from 6 to 20. At a noise variance level of 20, the position bias reaches approximately 39 m. Similarly, for speed bias, when the noise variance level is not greater than 0, the velocity bias is less than 1 m/s. The velocity bias changes less when the noise variance level is from −20 to 0. The velocity bias is greater than 1 m/s when the noise variance level is greater than 0. The position bias varies more when the noise variance level is from 0 to 20. At a noise variance level of 20, the velocity bias reaches about 15 m/s.

When the noise variance level is not greater than 2, the position RMSE remains below 5 m. In the range of −20 to 2, the position RMSE is comparable to the CRLB. However, for noise variance levels greater than 2 and from 2 to 20, the position RMSE diverges significantly from the CRLB. At a noise variance level of 20, the position RMSE reaches approximately 108 m, whereas the CRLB is around 27 m. For the velocity RMSE, when the noise variance level is not greater than −4, it remains below 1 m/s. From −20 to −4, the velocity RMSE shows minimal deviations from the CRLB. However, when the noise variance level exceeds −4 and ranges from −4 to 20, the velocity RMSE diverges more significantly from the CRLB. At a noise variance level of 20, the velocity RMSE reaches about 230 m/s, while the CRLB is approximately 13 m/s.

**Figure 10:**
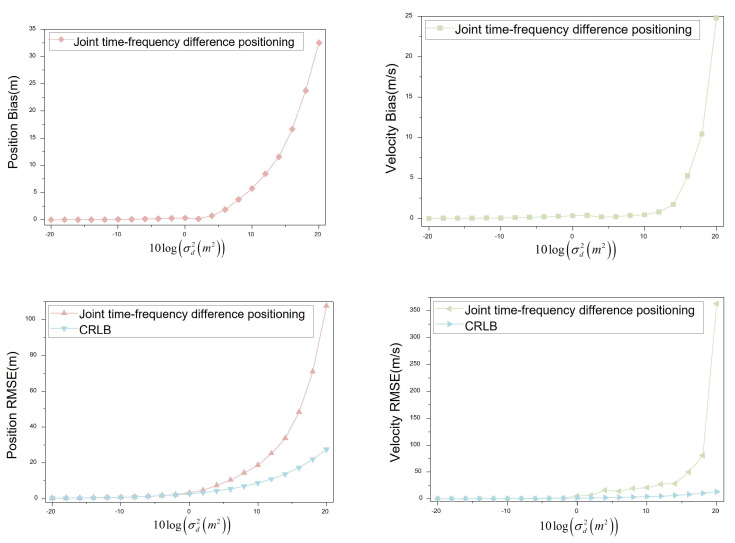
Positioning bias and RMSE (${\dot{R}}_{1}\left(20,0,0\right)$).

When the moving speed of the interference source is set to ${\dot{R}}_{1}\left(20,0,0\right)$, indicating the interference source moves horizontally at a speed of 20 m/s, the four UAVs conduct aerial monitoring at the speeds specified in Table 2. The obtained simulation data was visualized to analyze the positioning bias and RMSE variation trends, as shown in Figure 10.

The speed of the interference source is ${\dot{R}}_{1}\left(20,0,0\right)$). The position bias remains below 1 m when the noise variance level is not greater than 4. For noise variance levels ranging from −20 to 4, the position bias shows minimal changes. However, the position bias is greater than 1 m when the noise variance level is greater than 4. The position bias changes more when the noise variance level is from 6 to 20. At a noise variance level of 20, the position bias reaches approximately 33 m. Similarly, for speed bias, when the noise variance level is not greater than 12, the velocity bias is less than 1 m/s. The velocity bias changes less when the noise variance level is from −20 to 12. The velocity bias is greater than 1 m/s when the noise variance level is greater than 12. The position bias varies more when the noise variance level is from 12 to 20. At a noise variance level of 20, the velocity bias reaches about 25 m/s.

When the noise variance level is not greater than 2, the position RMSE remains below 5 m. In the range of −20 to 2, the position RMSE is comparable to the CRLB. However, for noise variance levels greater than 2 and from 2 to 20, the position RMSE diverges significantly from the CRLB. At a noise variance level of 20, the position RMSE reaches approximately 108 m, whereas the CRLB is around 28 m. For the velocity RMSE, when the noise variance level is not greater than −2, it remains below 1.5 m/s. From −20 to −2, the velocity RMSE shows minimal deviations from the CRLB. However, when the noise variance level exceeds −2 and ranges from −2 to 20, the velocity RMSE diverges more significantly from the CRLB. At a noise variance level of 20, the velocity RMSE reaches about 363 m/s, while the CRLB is approximately 13 m/s.

**Figure 11:**
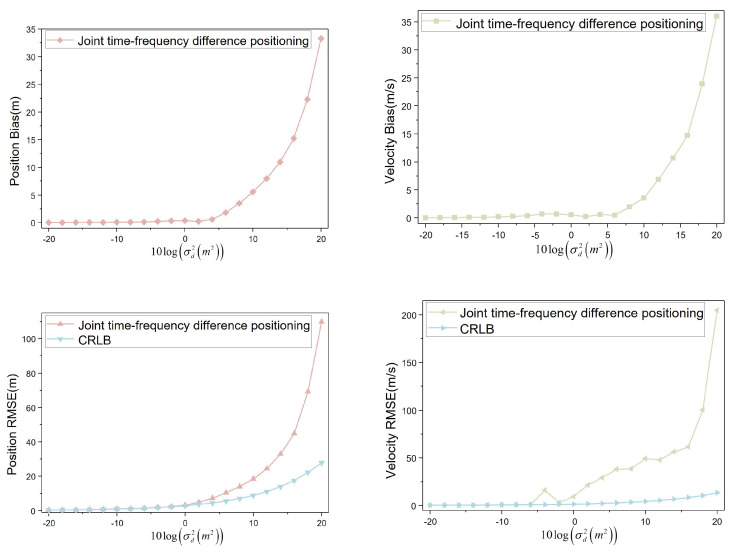
Positioning bias and RMSE (${\dot{R}}_{1}\left(40,0,0\right)$).

When the moving speed of the interference source is set to ${\dot{R}}_{1}\left(40,0,0\right)$, indicating that the interference source moves horizontally at a speed of 40 m/s, the four UAVs conduct aerial monitoring at the speeds specified in Table 2. The obtained simulation data were visualized to analyze the positioning bias and RMSE variation trends, as shown in Figure 11.

The speed of the interference source is ${\dot{R}}_{1}\left(40,0,0\right)$). The position bias remains below 1 m when the noise variance level is not greater than 4. For noise variance levels ranging from −20 to 4, the position bias shows minimal changes. However, the position bias is greater than 1 m when the noise variance level is greater than 4. The position bias changes more when the noise variance level is from 6 to 20. At a noise variance level of 20, the position bias reaches approximately 33 m. Similarly, for speed bias, when the noise variance level is not greater than 6, the velocity bias is less than 1 m/s. The velocity bias changes less when the noise variance level is from −20 to 6. The velocity bias is greater than 2 m/s when the noise variance level is greater than 8. The position bias varies more when the noise variance level is from 8 to 20. At a noise variance level of 20, the velocity bias reaches about 36 m/s.

When the noise variance level is not greater than 2, the position RMSE remains below 5 m. In the range of −20 to 2, the position RMSE is comparable to the CRLB. However, for noise variance levels greater than 4 and from 4 to 20, the position RMSE diverges significantly from the CRLB. At a noise variance level of 20, the position RMSE reaches approximately 110m, whereas the CRLB is around 28 m. For the velocity RMSE, when the noise variance level is not greater than −6, it remains below 1 m/s. From −20 to −6, the velocity RMSE shows minimal deviations from the CRLB. However, when the noise variance level exceeds −2 and ranges from −2 to 20, the velocity RMSE diverges more significantly from the CRLB. At a noise variance level of 20, the velocity RMSE reaches about 204 m/s, while the CRLB is approximately 13 m/s.

**Figure 12:**
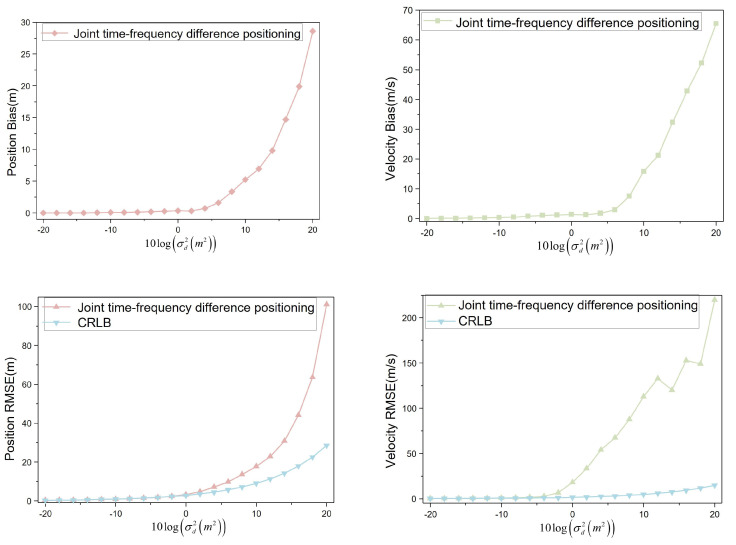
Positioning bias and RMSE (${\dot{R}}_{1}\left(80,0,0\right)$).

When the moving speed of the interference source is set to ${\dot{R}}_{1}\left(80,0,0\right)$, indicating the interference source moves horizontally at a speed of 80 m/s, the four UAVs conduct aerial monitoring at the speeds specified in Table 2. The obtained simulation data were visualized to analyze the positioning bias and RMSE variation trends, as shown in Figure 12.

The speed of the interference source is ${\dot{R}}_{1}\left(80,0,0\right)$). The position bias remains below 1 m when the noise variance level is not greater than 4. For noise variance levels ranging from −20 to 4, the position bias shows minimal changes. However, the position bias is greater than 1 m when the noise variance level is greater than 4. The position bias changes more when the noise variance level is from 6 to 20. At a noise variance level of 20, the position bias reaches approximately 28 m. Similarly, for speed bias, when the noise variance level is not greater than 6, the velocity bias is less than 3 m/s. The velocity bias changes less when the noise variance level is from −20 to 6. The velocity bias is greater than 7 m/s when the noise variance level is greater than 8. The position bias varies more when the noise variance level is from 8 to 20. At a noise variance level of 20, the velocity bias reaches about 65 m/s.

When the noise variance level is not greater than 2, the position RMSE remains below 5 m. In the range of −20 to 2, the position RMSE is comparable to the CRLB. However, for noise variance levels greater than 4 and from 4 to 20, the position RMSE diverges significantly from the CRLB. At a noise variance level of 20, the position RMSE reaches approximately 101 m, whereas the CRLB is around 28 m. For the velocity RMSE, when the noise variance level is not greater than −4, it remains below 2.5 m/s. From −20 to −4, the velocity RMSE shows minimal deviations from the CRLB. However, when the noise variance level exceeds −2 and ranges from −2 to 20, the velocity RMSE diverges more significantly from the CRLB. At a noise variance level of 20, the velocity RMSE reaches about 220 m/s, while the CRLB is approximately 15 m/s.

Based on the comparative analysis above, the following conclusions can be drawn: The velocity of the interference source in the horizontal direction ranges from 0 m/s to 80 m/s when considering certain initial positions and velocities for the UAV platform and the initial position of the interference source. For position estimation, when the noise variance level is below 4, the position bias remains below 1m. At a noise variance level of 20, the position bias ranges from 29 m to 39 m. For the RMSE of position, at noise variance levels below 2, it remains below 5 m. At a noise variance level of 20, the RMSE of the position ranges from 101 m to 110 m. These findings indicate that the movement velocity of the interference source has little effect on the positioning location results. On the other hand, the velocity of the interference source has a more significant impact on the positioning velocity results. The velocity bias remains below 1 m/s for different noise variance levels. At a noise variance level of 20, the velocity bias ranges from 15 m/s to 65 m/s. For the RMSE of velocity, when the noise variance level is below −6, it remains below 1 m/s and shows similarity to the CRLB. At a noise variance level of 20, the RMSE of velocity ranges from 204 m/s to 363 m/s, while the CRLB is approximately 13 m/s.

## 5. Conclusions

In this paper, through a combination of theoretical analysis, simulations, and other technical means, we conducted a study of the civil aviation radio interference source positioning method using four UAVs. Our study encompassed the design of time synchronization and communication for the four UAVs, the model of locating civil aviation radio interference sources by four UAVs, and the simulation of the positioning performance of this model. The simulation results show that the positioning performance of the four UAVs’ star-based deployment configuration is balanced and the positioning error is small, and the interference source movement velocity has a small impact on the positioning location accuracy and a large impact on the positioning velocity accuracy. The simulation results provide data support for the next experiments.

## 6. Outlook

The application of civil aviation radio interference source positioning based on multiple UAVs is still in the development stage, and limited by time, experimental equipment, experimental conditions, personal ability, and other factors, this paper is not perfect. Therefore, the following points are now proposed to carry out in-depth research in the following work.

(1) In order to be able to apply multi-UAV localized radio interference source equipment in practice, it is also necessary to integrate multi-UAV collaboration techniques, including UAV formation, UAV obstacle avoidance, and integration of all ground-based software into a single system.

(2) This paper does not consider the atmospheric refractive index error of the radio signals received by UAVs for the time being, and in order to improve the positioning accuracy, the empirical model of atmospheric refractive index can be incorporated into the multi-UAV positioning algorithm.

## Figures and Tables

**Table 1:** Cartesian coordinates of UAVs under different deployment configurations.

Deployment Configurations	$x_{i}$ (km)	$y_{i}$ (km)	$z_{i}$ (km)
Star deployment configuration UAV1	0	0	0.1
Star deployment configuration UAV2	−17	10	0.1
Star deployment configuration UAV3	17	10	0.1
Star deployment configuration UAV4	0	−20	0.1
Flat rhombus deployment configuration UAV1	0	0	0.1
Flat rhombus deployment configuration UAV2	−17	10	0.1
Flat rhombus deployment configuration UAV3	17	10	0.1
Flat rhombus deployment configuration UAV4	0	20	0.1
Inverted triangle deployment configuration UAV1	0	0	0.1
Inverted triangle deployment configuration UAV2	−20	20	0.1
Inverted triangle deployment configuration UAV3	20	20	0.1
Inverted triangle deployment configuration UAV4	0	20	0.1
Parallelogram deployment configuration UAV1	0	0	0.1
Parallelogram deployment configuration UAV2	−14	14	0.1
Parallelogram deployment configuration UAV3	14	14	0.1
Parallelogram deployment configuration UAV4	28	0	0.1

**Table 2:** Coordinates and speed information of four UAVs.

UAV	$x_{i}$ (m)	$y_{i}$ (m)	$z_{i}$ (m)	${\dot{x}}_{i}$ (m/s)	${\dot{y}}_{i}$ (m/s)	${\dot{z}}_{i}$ (m/s)
$U_{1}$	290	100	150	20	−20	20
$U_{2}$	380	150	100	−20	10	20
$U_{3}$	300	490	200	10	−20	10
$U_{4}$	340	200	90	10	20	30

## Data Availability

The data that support the findings of this research are available from the author X.Z. upon reasonable request.
